# Primary outcome reporting in clinical trials for older adults with depression

**DOI:** 10.1192/bjo.2023.650

**Published:** 2024-03-07

**Authors:** Myanca Rodrigues, Anna Oprea, Keily Johnson, Alexander Dufort, Nitika Sanger, Pegah Ghiassi, Stephanie Sanger, Balpreet Panesar, Alessia D'Elia, Sameer Parpia, Zainab Samaan, Lehana Thabane

**Affiliations:** Health Research Methodology Graduate Program, Department of Health Research Methods, Evidence, and Impact, McMaster University, Canada; Life Sciences Undergraduate Program, School of Interdisciplinary Science, McMaster University, Canada; Psychology, Neuroscience and Behaviour Undergraduate Program, Faculty of Science, McMaster University, Canada; Department of Psychiatry and Behavioural Neurosciences, McMaster University, Canada; Delivery Management Office, Canadian Partnership Against Cancer, Toronto, Canada; Health Sciences Library, McMaster University, Canada; Neuroscience Graduate Program, McMaster University, Canada; and Department of Psychiatry and Behavioural Neurosciences, St. Joseph's Healthcare Hamilton, Ontario, Canada; Department of Oncology, McMaster University, Canada; and Department of Health Research Methods, Evidence, and Impact, McMaster University, Canada; Department of Psychiatry and Behavioural Neurosciences, McMaster University, Canada; Department of Health Research Methods, Evidence, and Impact, McMaster University, Canada; and Mood Disorders Program, St. Joseph's Healthcare Hamilton, Ontario, Canada; Department of Health Research Methods, Evidence, and Impact, McMaster University, Canada; Population Health Research Institute, Ontario, Canada; and Father Sean O'Sullivan Research Centre, St. Joseph's Healthcare Hamilton, Ontario, Canada

**Keywords:** Depressive disorders, clinical outcome measures, older adults, outcome reporting, randomised controlled trials

## Abstract

**Background:**

Findings from randomised controlled trials (RCTs) are synthesised through meta-analyses, which inform evidence-based decision-making. When key details regarding trial outcomes are not fully reported, knowledge synthesis and uptake of findings into clinical practice are impeded.

**Aims:**

Our study assessed reporting of primary outcomes in RCTs for older adults with major depressive disorder (MDD).

**Method:**

Trials published between 2011 and 2021, which assessed any intervention for adults aged ≥65 years with a MDD diagnosis, and that specified a single primary outcome were considered for inclusion in our study. Outcome reporting assessment was conducted independently and in duplicate with a 58-item checklist, used in developing the CONSORT-Outcomes statement, and information in each RCT was scored as ‘fully reported’, ‘partially reported’ or ‘not reported’, as applicable.

**Results:**

Thirty-one of 49 RCTs reported one primary outcome and were included in our study. Most trials (71%) did not fully report over half of the 58 checklist items. Items pertaining to outcome analyses and interpretation were fully reported by 65% or more of trials. Items reported less frequently included: outcome measurement instrument properties (varied from 3 to 30%) and justification of the criteria used to define clinically meaningful change (23%).

**Conclusions:**

There is variability in how geriatric depression RCTs report primary outcomes, with omission of details regarding measurement, selection, justification and definition of clinically meaningful change. Outcome reporting deficiencies may hinder replicability and synthesis efforts that inform clinical guidelines and decision-making. The CONSORT-Outcomes guideline should be used when reporting geriatric depression RCTs.

Randomised controlled trials (RCTs) are often deemed the gold standard in comparative effectiveness research, since their synthesis through systematic reviews and meta-analyses is used to inform clinical care guidelines that guide evidence-informed practice.^1^ However, inconsistency and insufficiency in reporting of clinical trials, and in particular, their outcomes, is a long-standing issue in biomedical research, and challenges evidence-based care.^2–8^ Outcomes or end-points indicate intervention success or effectiveness, and are essential components of clinical trials.^6,9,10^ However, prior research has demonstrated that clinical trials insufficiently report the rationale for outcome selection, definition of the outcome, outcome measurement details and methodology for outcome analysis.^3,5,11–13^ Deficiencies in outcome reporting in trials (i.e. lack of sufficient details reported to ensure complete understanding of the end-point) impedes the reproducibility of trials and cross-study comparison of results, and further limits the uptake of research to clinical practice, thereby contributing to research waste.^14–17^ Although prior research has examined primary outcome reporting in trials for adolescents with major depressive disorder (MDD),^18^ reporting comprehensiveness of primary outcomes has not been assessed in RCTs for geriatric depression.

## Outcome reporting in geriatric depression trials

Depression is one of the leading causes of disability for older adults worldwide, accounting for an estimated loss of 13.8 years of quality-adjusted life expectancy at 65 years of age.^19^ Adverse health outcomes for this clinical population often include a reduced quality of life,^20^ disability^21^ and mortality.^22^ Geriatric MDD is often treated with one or a combination of interventions including, but not limited to, pharmacotherapy,^23^ psychotherapy^24^ and exercise therapy.^25^ However, there is still uncertainty regarding intervention effectiveness for this unique clinical population, given the prevalence of comorbid mental and physical illnesses that often accompany aging,^23^ and must be considered during selection of the treatment course because of potential drug–drug interactions between antidepressants and concomitant medications.^23^ The uncertainty in assessing intervention effectiveness may be partially attributed to variability in outcome reporting and subsequent challenges in interpretation and synthesis of trial findings, which impedes clinical decision-making for geriatric depression. Previous meta-analyses of pharmacological^26,27^ and psychosocial^28^ interventions for older adults with depression have reported limitations in interpretability of findings as a result of the heterogeneity in the use of outcomes across trials.

Our recent review identified substantial variability in the outcomes reported by RCTs.^29^ Additionally, up to 19 outcome measurement instruments (OMIs) were used to measure the single outcome, ‘depressive symptom severity’.^29^ Although prior meta-analyses suggest variability in outcome measurement and descriptions,^26–28^ there has not been a systematic assessment of outcome reporting comprehensiveness for geriatric depression. A thorough assessment of the comprehensiveness of outcome reporting in trials is integral to understanding the presence and extent of the issue, and inform the need for standardising outcome reporting in trials assessing older adults with MDD. The objective of our study is to extend our previous work, and assess the comprehensiveness of primary outcome reporting in published geriatric depression trials.

## Method

### Study selection

This study is registered with the International Prospective Register of Systematic Reviews (PROSPERO; registration number: CRD42021244753). Our study was conducted in conjunction with a systematic survey to identify eligible trials.^29^ We included RCTs assessing any type of intervention for unipolar, non-psychotic MDD for adults aged 65 years and older, which were published in English between 1 January 2011 and 16 July 2021 inclusive. Trials evaluating people with comorbid mental disorders including depression, and those that presented a subgroup analysis containing adults aged 65 years and older, were also included. Pilot and feasibility trials, and follow-up studies and secondary analyses, were included when the primary RCT was published outside of our timeframe. The protocol for this study, which contains detailed search strategy and eligibility criteria, has been published.^30^ In summary, we searched Medline, EMBASE, PsycINFO and the Cochrane Central Register of Controlled Trials databases to identify eligible trials. Title/abstract and full-text screening was conducted independently and in duplicate, using Covidence systematic review software.^31^ We supplemented our electronic search with a manual search for potentially eligible trials by reviewing the references of all included studies. Discrepancies regarding study inclusion resolved through discussion between reviewers, and a third reviewer, when necessary, to reach consensus during every stage of screening.

As our objective was to assess reporting comprehensiveness of primary outcomes, we restricted the sample to trials that specified a single, discernible primary outcome. Thus, for our present study, two reviewers applied additional eligibility criteria, independently and in duplicate. Specifically, these trials either (a) explicitly described these outcomes as ‘primary’ or using an appropriate synonym; (b) stated that the study aimed to examine the effect of an intervention on that specific outcome in the objectives or (c) used data from that outcome to power the sample size for the trial.^32^ Studies with multiple primary outcomes, and/or those for which a primary outcome was not clearly stated, were therefore excluded from our present study as the primary outcome could not be inferred. For pilot and feasibility studies, which are conducted in preparation for full-scale RCTs and also include outcomes pertaining to feasibility,^33^ we solely considered effectiveness outcomes, in concordance with the objectives of our systematic survey.^29^

### Assessment of outcome reporting

We assessed the comprehensiveness of primary outcome reporting for trials included in our study by using a checklist of 70 outcome reporting items. These items were also used by a previous study to evaluate comprehensiveness of outcome reporting in adolescent depression trials,^34^ and in the development of the Consolidated Standards of Reporting Trials (CONSORT)-Outcomes checklist (an essential set of reporting items to be included for primary and secondary trial outcomes in published trials).^35^ The CONSORT-Outcomes checklist is an extension of the CONSORT 2010 statement (a minimum, recommended set of items to be reported by RCTs).^36^ The 70-item checklist used in our study and the CONSORT-Outcomes checklist both contain outcome reporting items that spanned the following thematic categories: (a) who (source of information for the outcome), (b) what (outcome description), (c) where (location and setting of outcome assessment), (d) when (timing of outcome measurement), (e) why (rationale for outcome selection), (f) how (method of outcome measurement), (g) management and analysis of outcome data, (h) missing outcome data, (i) outcome interpretation and (j) any modifications made to the outcome.^35,37–39^

Of the 70 items, we found 12 items to be irrelevant or unable to be assessed in our study. These items are detailed with reasons for exclusion in Table 2. Thus, the outcome reporting assessment was conducted with the resulting 58-item checklist, similar to the assessment of primary outcome reporting across adolescent depression trials.^34^ Study team members (A.O., K.J.) were trained by a methodologist (M.R.) before conducting assessment of outcome reporting, using a sample of three randomly selected RCTs (see Supplementary File 1 available at https://doi.org/10.1192/bjo.2023.650 for the training guide). Once consensus was reached (≥80% agreement between reviewers) for each of the three trials, outcome reporting assessment was conducted for other studies independently and in duplicate, using predefined standardised data charting forms on Microsoft Excel (Microsoft Corporation; see https://office.microsoft.com/excel) from 31 January 2023 to 31 March 2023. Any disagreements were resolved through discussion, and by a third reviewer (M.R.) as needed to reach consensus. We used the same assessment process for every trial included in our study, in order to reach consensus on all appraised items.

### Scoring details

We assessed outcome reporting for each of the 58 checklist items as ‘fully reported’, ‘partially’ reported’ or ‘not reported’ for the primary outcome in every trial. A score of ‘fully reported’ was given to items where full details for the item were reported by included studies. This included instances where previously published supplementary materials (i.e. protocols, statistical analysis plans or other reports) were referenced by the authors regarding a particular reporting item. Conversely, items which were ‘partially reported’ by trials reported one or a few items of a multi-component item. This classification only applied to checklist items comprising multiple components (see Table 2 for list), i.e. item 23 (reliability of the OMI in a similar study setting). For instance, this item was scored as ‘partially reported’ when authors indicated that the OMI was reliable but did not specify whether reliability was established in a similar study setting. If no information was provided for the item, or the concept of the particular item was irrelevant to the particular trial based on the information provided in the study, items were classified as ‘not reported’ or ‘not applicable’, respectively. For instance, if the trial did not report having missing data, item 52 was scored as ‘not reported’, and item 55 (justification for methods used to handle missing data) was subsequently deemed ‘not applicable’.

### Synthesis of findings

Study characteristics and results for reporting items were analysed descriptively with counts and frequencies. Outcome reporting comprehensiveness was calculated for each trial as a composite measure based on the percentage of items assessed as ‘fully reported, ‘partially reported’ and ‘not reported’.

## Results

### Search results

**Fig. 1:**
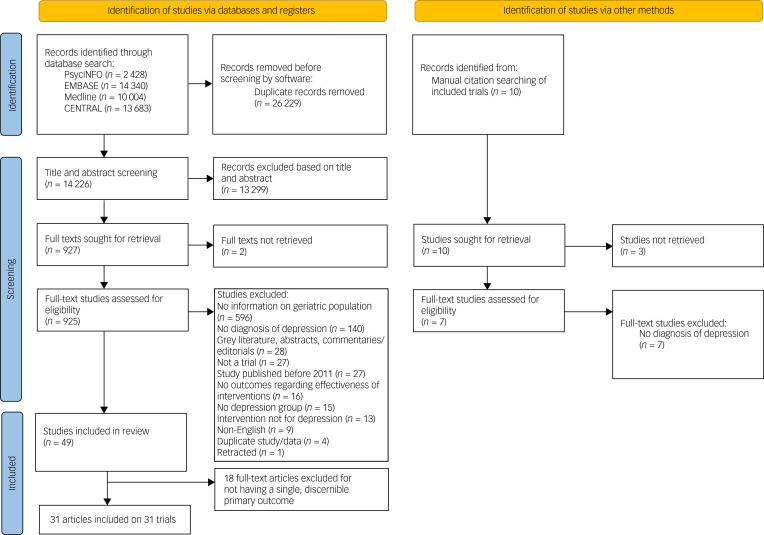
Preferred Reporting Items for Systematic Reviews and Meta-Analyses (PRISMA) flow diagram for trials assessing treatment interventions for major depressive disorder in older adults.

We identified 49 RCTs with the initial eligibility criteria, and excluded 18 trials for not having a single, discernible primary outcome. Our current study includes 31 RCTs; 22 studies (71%) explicitly deemed an outcome as ‘primary’, six (19%) aimed to assess the effect of an intervention on that particular end-point and three (10%) used data from the outcome to power the sample size for the trial (see Fig. 1 for the flow diagram^40^). Our complete dataset may be found in Supplementary File 2, with references to all included trials in Supplementary File 3.

### Characteristics of included trials

The characteristics of the 31 RCTs included in our study are described in Table 1. Most included studies were conducted in Europe (number of studies *k* = 11, 36%) or North America (*k* = 8, 26%), with the majority being publicly funded (*k* = 16, 52%). Nearly half the trials assessed pharmacological interventions (*k* = 15, 48%), with the remainder of studies assessing psychosocial (*k* = 10, 32%), case management (*k* = 5, 16%) or acupressure (*k* = 1, 3%) interventions. The number of participants in included studies ranged from 13 to 1879, with a median sample size of 174. The most commonly reported primary outcome was ‘depressive symptom severity’, reported by 15 trials (48%), followed by ‘depression treatment response’ (*k* = 12, 39%; see Supplementary Table 1(a) for definitions and frameworks used to classify outcomes in our original study).

**Table 1:** Characteristics and primary outcomes of included studies

Study	Intervention type	Age cut-off for included population, years^a^	Total sample size at enrolment	Length of follow-up (weeks)	Region of study	Funding source^b^	Primary outcome
Primary randomised clinical trials							
Banerjee et al., 2011	Drug	Not reported	326	39	Europe	Public	Depression treatment response
Brody et al., 2011	Drug	Not reported	16	16	North America	Industry	Depression treatment response
Chen et al., 2011	Drug	≥65	55	8	Asia	Not reported	Depression treatment response
Rondanelli et al., 2011	Drug	65–95	46	52	Europe	Public	Depressive symptom severity
Tajalizadekhoob et al., 2011	Drug	≥65	66	26	Asia	Academic	Depressive symptom severity
Cheng et al., 2012	Psychosocial	Not reported	36	12	Asia	Public	Depressive symptom severity
Fields et al., 2012	Drug	≥70	449	260	North America	Public	Depressive symptom severity
Preschl et al., 2012	Psychosocial	≥65	36	13	Europe	Not reported	Depression treatment response
Heun et al., 2013	Drug	≥65	222	8	Europe/North America/South America	Public	Depressive symptom severity
Katila et al., 2013	Drug	≥66	338	12	Europe/North America/South America	Industry	Depression treatment response
Robinson et al., 2014	Drug	≥65	370	24	Europe/North America	Industry	Depression treatment response
Bruce et al., 2015	Management	≥65	306	52	North America	Public	Depression treatment response
Imai et al., 2015	Psychosocial	≥65	184	34	Asia	Academic	Depressive symptom severity
McMusker et al., 2015^c^	Psychosocial	≥65	38	26	North America	Public	Depressive symptom severity
van Beljouw et al., 2015	Psychosocial	≥65	263	104	Europe	Public	Depressive symptom severity
Aakhus et al., 2016	Management	≥65	385	60	Europe	Foundation/non-profit plus public	Provider treatment adherence
Bosanquet et al., 2017	Management	≥65	485	78	Europe	Public	Depressive symptom severity
Hummel et al., 2017	Psychosocial	≥65	155	17	Europe	Not reported	Depressive symptom severity
Chang et al., 2018	Psychosocial	Not reported	93	12	Asia	Public	Depression treatment response
Emsley et al., 2018	Drug	≥65	311	8	Asia/Europe/North America	Public	Depression treatment response
Molassiotis et al., 2020	Acupressure	≥65	118	13	Asia	Academic	Depressive symptom severity
Ochs-Ross et al., 2020	Drug	≥65	137	10	Not reported	Industry	Depression treatment response
Berk et al., 2021	Drug	≥65	1879	21	Australia/New Zealand	Public	Depressive symptom severity
Pilot and feasibility studies							
Tomasino et al., 2017	Psychosocial	≥65	47	8	North America	Public	Depressive Symptom Severity
Burroughs et al., 2019^d^	Management	≥65	38	17	Europe	Public	Depression treatment response
Bollmann et al., 2020	Psychosocial	≥65	13	4	Europe	Not reported	Depressive symptom severity
Follow-up studies and secondary analyses							
Azar et al., 2011	Management	≥65	792	26	North America	Academic	Depression remission
Drye et al., 2011	Drug	Not reported	131	24	North America	Public	Depressive symptom severity
Holman et al., 2011^e^	Psychosocial	≥65	204	43	Europe	Foundation/non-profit	Cost-effectiveness of study interventions
Dolberg et al., 2014	Drug	≥65	405	36	Europe	Not reported	Depression relapse
Zilcha-Mano et al., 2018	Drug	≥75	174	8	North America	Public	Depression treatment response

a. As the mean or median ages for the included populations in the majority of studies were unclear, we have indicated the age cut-offs.

b. Funding sources categorised as follows: public: funded by a governmental organisation (e.g. National Institute of Mental Health, National Institute for Health Research); industry: for-profit corporation (e.g. Janssen Research & Development, AstraZeneca Pharmaceuticals); academic: university or other academic institution (e.g. Harvard Medical School, Tehran University of Medical Science); not for profit: not-for-profit foundation or organisation (e.g. The Health Foundation).

c. Study included both younger populations and older adults with major depressive disorder, but reported data stratified by age for those aged ≥65 years. Information has been extracted for this stratified population, which fulfilled our study inclusion criteria.

d. Feasibility trial.

e. Follow-up study.

### Outcome reporting assessment

**Fig. 2:**
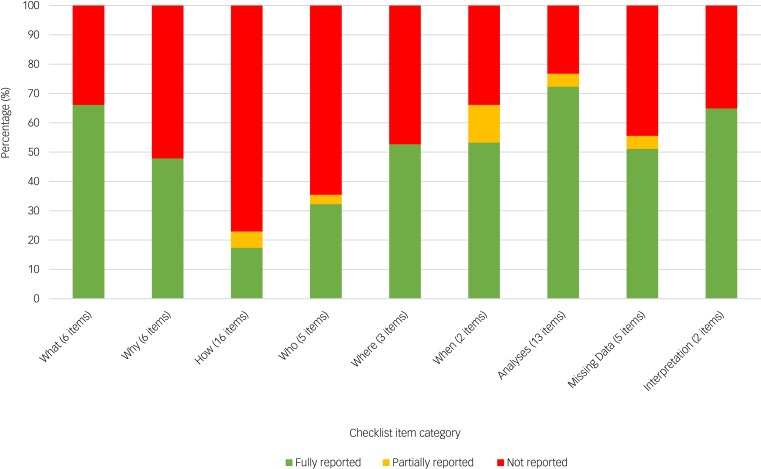
Outcome reporting comprehensiveness across 31 geriatric major depressive disorder trials, by thematic item category.

Overall, there was variation in the items scored as ‘fully reported’, ‘partially reported’ or ‘not reported’ across the thematic categories (Fig. 2). The category ‘Outcome data management and analyses’ had the highest percentage of fully reported items (73%), followed by ‘What: Description of the outcome’ (66%) and ‘Outcome interpretation’ (65%). The lowest percentage of fully reported items were observed for the categories ‘How: Method of outcome measurement’ (17%) and ‘Who: Source of information for the outcome’ (32%).

**Fig. 3:**
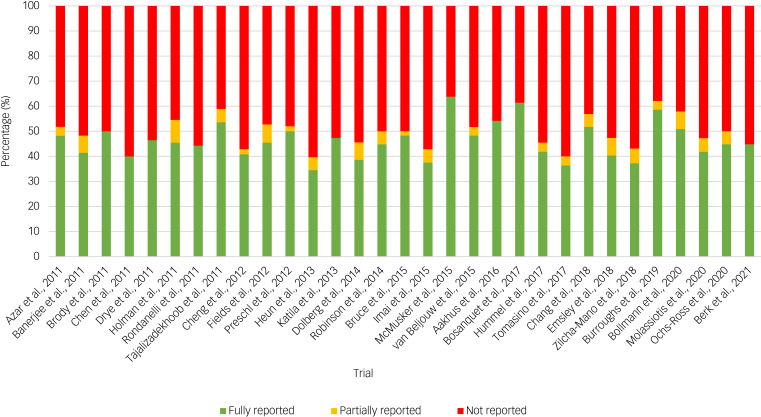
Outcome reporting comprehensiveness across 31 geriatric major depressive disorder trials.

The assessment of outcome comprehensiveness was variable for each of the included 31 RCTs. Overall, each study fully reported about half of the 58 checklist items (Fig. 3, Supplementary File 2). The percentage of items that were fully reported by each trial varied from 34 to 64%, with a median of 45%. The percentage of items that were fully reported remained relatively stable from 2011 through 2021, i.e. over a 10-year period (Fig. 3). We describe outcome reporting comprehensiveness for each thematic category in the following sections, with reporting frequencies for all 58 items presented in Table 2.

**Table 2:** Frequency of outcome reporting classifications for each reporting item for the primary outcome in included trials (*n* = 31)

Reporting item thematic category and number	Fully reported, *n* (%)	Partially reported, *n* (%)^a^	Not reported, *n* (%)
What: Description of the outcome			
1. Described the outcome domain^b^	31 (100)	Not applicable	0 (0)
2. Stated the outcome	31 (100)	Not applicable	0 (0)
[Item excluded from reporting assessment]: If reporting a composite outcome (i.e. two or more component outcomes that are combined), all individual components are defined^c^	There were no composite outcomes in the sample of included studies, so this item did not apply		
[Item excluded from reporting assessment]: If there are other published definitions of the outcome, explained why the chosen definition was used^c^	There was no indication in the included studies that other published definitions of the outcome exist, so this item did not apply		
3. Specified the outcome as primary	31 (100)	Not applicable	0 (0)
4. Provided a rationale for classifying the outcome as primary, instead of secondary	7 (23)	Not applicable	24 (77)
5. Defined clinical significance on the outcome (e.g. minimal important difference, responder definition), including what would constitute a good or poor outcome	16 (52)	Not applicable	15 (48)
6. Justified the criteria used for defining meaningful change (e.g. the minimal important difference, responder definition), including what would constitute a good or poor outcome, such as from an outcome measurement interpretation guideline	7 (23)	Not applicable	24 (77)
Why: Rationale for selecting the outcome			
7. Explained how the outcome addresses/relates to the hypothesis of the study	27 (87)	Not applicable	4 (13)
8. Explained how the outcome addresses the objective/research question of the study (i.e. to compare the effect of intervention A versus intervention B on outcome X)	31 (100)	Not applicable	0 (0)
[Item excluded from reporting assessment]: Specified if the outcome is part of a core outcome set, if a core outcome set is publicly available. If so, refer to which core outcome set it is part of (e.g. via www.comet-initative.org/)^c^	There is currently no core outcome set for geriatric depression, so this item did not apply		
[Item excluded from reporting assessment]: If completely new outcome, justified why other outcomes are not appropriate or relevant^c^	Included trials made no note that the reported primary outcome is completely new; thus, this item did not apply		
9. Described why the outcome is relevant to each stakeholder group involved in this trial (e.g. patients, decision makers, policy makers, clinicians, funders, etc.)	2 (6)	Not applicable	29 (94)
10. Reported which stakeholders (e.g. patients, decision makers, policy makers, clinicians, funders, etc.) were actively involved in outcome selection (this should be documented as per recent guidance for the reporting of patient and public involvement)	2 (6)	Not applicable	29 (94)
11. Explained the mechanism (e.g. pathophysiological, pharmacological, etc.) or theoretical framework/model by which the experimental intervention is expected to cause change in the outcome in the target population	21 (68)	Not applicable	10 (32)
12. Provided rationale for the choice of the specific type of outcome (i.e. why a patient-reported outcome instead of a clinician reported outcome)	6 (19)	Not applicable	25 (81)
How: The way the outcome is measured			
13. Described the outcome measurement instrument used. This should include instrument scaling and scoring details (e.g. range and direction of scores)^d^	15 (48)	16 (52)	0 (0)
14. Specified whether the outcome measurement instrument will be used in accordance with any user manual and specify and justify deviations if planned	6 (19)	Not applicable	25 (81)
15. Specified a recall period for outcome assessment, if applicable	0 (0)	Not applicable	31 (100)
16. Described mode of outcome assessment (e.g. face to face, telephone, electronically)	18 (58)	Not applicable	13 (42)
17. Justified the mode of outcome assessment (e.g. justification for equivalence between different modes of administration, if applicable)	6 (19)	Not applicable	25 (81)
18. Described any additional resources/materials or processes when performing outcome assessment, when relevant (i.e. a stethoscope, language interpreter, fasting before a colonoscopy, etc.)	2 (6)	Not applicable	29 (94)
19. Described or provided reference to an empirical study that established validity of the outcome measurement instrument in individuals similar to the study sample (i.e. measures what it is supposed to measure)^d,e^	9 (30)	4 (13)	17 (57)
20. Described or provided reference to an empirical study that established validity of the outcome measurement instrument in the study setting (i.e. measures what it is supposed to measure)^d,e^	5 (17)	2 (7)	23 (77)
21. Specified whether more than one language version of the outcome measurement instrument was used and state whether translated versions have been developed using currently recommended methods, if applicable^e^	5 (17)	0 (0)	25 (83)
22. Described or provided reference to an empirical study that established reliability of the outcome measurement instrument in individuals similar to the study sample (i.e. ability to produce consistent results)^d,e^	4 (13)	1 (3)	25 (83)
23. Described or provided reference to an empirical study that established reliability of the outcome measurement instrument in the study setting (i.e. ability to produce consistent results)^d,e^	2 (7)	0 (0)	28 (93)
24. Described or provided reference to an empirical study that established the responsiveness of the outcome measurement instrument in the study sample (i.e. ability to detect change over time given a change in disease activity or status)^e^	3 (10)	0 (0)	27 (90)
25. Described the feasibility of the outcome measurement instrument in the study sample (i.e. the practical considerations of using an instrument, including its ease of use, time to complete, monetary costs and interpretability of the question(s) included in the instrument)^e^	2 (7)	1 (3)	27 (90)
26. Described the acceptability and burden of the outcome measurement instrument in the study sample^d, e^	1 (3)	0 (0)	29 (97)
27. Specified whether order of administration of outcome measurement instrument was standardised, if assessing multiple outcomes^e^	1 (4)	0 (0)	26 (96)
28. Specified whether or not outcome data will be monitored during the study to inform the clinical care of individual trial participants, and if so, how this will be managed in a standardised way	5 (16)	3 (10)	23 (74)
Who: Source of information of the outcome			
29. Described who (e.g. nurse, occupational therapist, technician, parent, outcome adjudicators), and if applicable, how many assessed the outcome in each study group	16 (52)	5 (16)	10 (32)
30. Justified the choice of outcome assessor(s) (e.g. proxy versus healthcare provider)	3 (10)	Not applicable	28 (90)
31. Described whether the outcome assessor(s) were blinded/masked to intervention assignment	19 (61)	Not applicable	12 (39)
32. Described any trial-specific training required for outcome assessors to apply the outcome measurement instrument	8 (26)	Not applicable	23 (74)
33. Described how outcome data quality was maximised (e.g. duplicate measurements)	4 (13)	Not applicable	27 (87)
Where: Assessment location and setting of the outcome			
[Item excluded from reporting assessment]: Specified the name, affiliation and contact details for the individual(s) responsible for the outcome content to identify the appropriate point of contact for resolution of any outcome-specific inquiries^c^	This item concept is not an outcome-level reporting item, but a trial-level reporting item, and therefore it is not pertinent for assessing the outcome reporting of the included trial reports		
34. Specified geographic location of outcome assessment for each study group (e.g. list of countries where outcome data was collected)	30 (97)	Not applicable	1 (3)
35. Described setting of outcome assessment for each study group (e.g. clinic, home, other)	19 (61)	Not applicable	12 (39)
36. Justified suitability of the outcome assessment setting(s) for the study sample (e.g. family doctor office versus home when measuring blood pressure)	0 (0)	Not applicable	31 (100)
When: Timing of measurement of the outcome			
37. Specified timing and frequency of outcome assessment(s) for outcomes (e.g. time point for each outcome, time schedule of assessments)^e^	31 (100)	0 (0)	0 (0)
38. Provided justification of timing and frequency of outcome assessment(s) (such as pathophysiological or epidemiological evidence for disease processes and complications to occur and/or pragmatic justification)	2 (6)	8 (26)	21 (68)
Outcome data management and analyses			
[Item excluded from reporting assessment]: Provided the results of all planned outcome analyses that were undertaken, regardless of statistical significance^c^	This item required access to the protocols or statistical analysis plans of the included studies (if publicly available), which was out of scope for the objectives of our study, and therefore not conducted		
39. Provided definition of outcome analysis population	31 (100)	Not applicable	0 (0)
40. Described unit of analysis of the outcome (i.e. cluster or individual)	31 (100)	Not applicable	0 (0)
41. Described outcome analysis metric (e.g. change from baseline, final value, time to event)	31 (100)	Not applicable	0 (0)
42. Described method of aggregation for outcome data (e.g. mean, median, proportion)	31 (100)	Not applicable	0 (0)
43. Described statistical methods and/or significance test(s) (name or type) used for analysing outcome data. This should include any analyses undertaken to address multiplicity/type 1 (α) error, particularly for trials with multiple domains and time points^d^	31 (100)	0 (0)	0 (0)
44. Described the covariates and factors in the statistical model (e.g. adjusted analyses) used for analysing outcome data, if applicable^e^	18 (100)	Not applicable	0 (0)
45. Provided justification for covariates and factors and why they were selected, if applicable^e^	4 (22)	Not applicable	14 (78)
46. Described results for each group, including estimated effect size and its precision (such as 95% confidence interval). For binary outcomes, presentation of both absolute and relative effect sizes is recommended^d^	26 (84)	5 (16)	0 (0)
47. Described time period (i.e. chronological time since randomisation) for which the outcome was analysed	31 (100)	Not applicable	0 (0)
[Item excluded from reporting assessment]: Described how unplanned repeat measurements were handled when analysing outcome data (e.g. repeat blood pressure result in patient because of initial abnormal reading)^c^	Included studies did not indicate that unplanned repeated measurements occurred, so this item did not apply		
48. Described outcome data, assessment process and analysis for participants who discontinued or deviated from the assigned intervention protocol^d^	9 (29)	10 (32)	12 (39)
49. Described outcome data entry, coding, security and storage, including any related processes to promote outcome data quality (e.g. double entry; range checks from outcome data values)	1 (3)	1 (3)	29 (94)
[Item excluded from reporting assessment]: If someone other than a member in the study group analysed the outcome data, described the person's affiliations (e.gif the person is affiliated with industry)^c^	Included studies did not indicate that someone outside the study group analysed the outcome data, so we could not assess this item		
50. Described blinding procedure(s) applied to data entry personnel and/or data analysts	6 (19)	0 (0)	25 (81)
[Item excluded from reporting assessment]: Describe any plans to minimise missing outcome data^c^	This item concept applies more so to outcome reporting in protocols of trials, and is therefore out of scope for outcome reporting of trials		
51. Described methods for additional analyses (e.g. subgroup analyses), if applicable^e^	11 (73)	Not applicable	4 (27)
Missing outcome data			
52. Described how much outcome data was missing^e^	26 (84)	Not applicable	5 (16)
53. Described any reasons for missing outcome data in each arm (i.e. reasons for withdrawal or reasons for lack of follow-up). Please provide enough detail that the reported reason can be used to reduce the uncertainty about the potential underlying mechanism of missing outcome data^e^	19 (73)	2 (8)	5 (19)
54. Explained statistical methods to handle missing outcome items or entire assessments (e.g. multiple imputation)^e^	12 (46)	1 (4)	13 (50)
55. Provided justification for methods used to handle missing outcome data. This should include: (a) assumptions underlying the missing outcome data mechanism with justification (including analyses performed to support assumptions about the missingness mechanism); and (b) how the assumed missingness mechanism and any relevant features of the outcome data would influence the choice of statistical method(s) to handle missing outcome data including sensitivity analyses^d,e^	4 (15)	3 (12)	19 (73)
56. Described any outcome analyses conducted to assess the risk of bias posed by missing outcome data (e.g. comparison of baseline characteristics of participants with and without missing outcome data)^e^	8 (31)	Not applicable	18 (69)
Outcome interpretation			
57. Interpret outcome data in relation to clinical outcomes, where relevant	31 (100)	Not applicable	0 (0)
58. Discussed impact of missing outcome data on the interpretation of findings, if applicable^e^	6 (23)	Not applicable	20 (77)
[Item excluded from reporting assessment]: Described other considerations or procedures that could affect the ability to interpret the outcome results (e.g. for per-protocol analysis, describe the limitations in the methods used to monitor subject adherence)^c^	The item concept is subjective in nature and was excluded, given that could not be assessed in a standardised way		
Modifications			
[Item excluded from reporting assessment]: Described any changes to trial outcomes after the trial commenced, with reasons^c^	Included studies did not note that changes to trial outcomes occurred, so we could not assess this item		
[Item excluded from reporting assessment]: Described if there were any changes made to the planned analysis of outcomes (including omissions) after the trial commenced, and if yes, provided justification. For example, if any prespecified covariates are omitted in the model, describe which covariates were omitted and justify the omissions, including any statistical methods employed to justify this omission^c^	Included studies did not note that changes to planned analysis of outcomes occurred, so we could not assess this item		

This table has been adapted from an assessment of primary outcome reporting in adolescent depression trials.^34^

a. Not applicable refers to instances where ‘partially reported’ was not a valid assessment option. Items scored as ‘Not applicable’ were not included in the overall scoring, since they were deemed to be irrelevant to the assessment of outcome reporting by the research team (M.R., A.O., K.J., L.T., S.P., Z.S.), by consensus.

b. Outcome domain defined in accordance with core taxonomic framework proposed by Dodd et al.^10,29^ Given that domains are broad and not directly measurable, outcomes are selected to assess change within them. See Supplementary Table 1(a) for further details.

c. Outcome reporting items removed from the comprehensive item checklist, and subsequently excluded from reporting assessment.

d. Item was considered ‘fully reported’ only when all components for that item were reported in the trial, e.g. for item 13, if both scaling and scoring details were reported.

e. Several items do not add to a total denominator of *N* = 31 trials for the following reasons: items 19–26 (denominator: 30 trials) were not applied to a trial where the primary outcome was behavioural change, i.e. change in provider treatment adherence, which does not have gold standard measures of validity, reliability, etc.; item 27 (denominator: 27 trials) did not apply to trials that assessed only one outcome; items 44 and 45 (denominator: 18 trials) were not assessed for trials that did not include covariates/factors in their statistical models; item 51 (denominator: 15 trials) only applied to trials that conducted additional analyses; and items 53–56 and 58 (denominator: 26 trials) were only applied to trials that reported having missing data.

### What: description of the outcome

Every included trial described the outcome domain, stated the outcome and specified the outcome as primary (*k* = 31, 100%; items 1–3, respectively). However, only 23% (*k* = 7/31) of included studies fully reported a rationale for classifying the outcome as primary (item 4). Although just over half (*k* = 16, 52%; item 5) of included RCTs defined clinical significance of the outcome, the criteria used to define meaningful change was infrequently reported by studies (*k* = 7, 23%; item 6).

### Why: rationale for selecting the outcome

There was variation in the descriptions of the rationale for outcome selection by trials included in our study. Outcome items that were most frequently reported included explanations of how the outcome addresses the research question (*k* = 31, 100%; item 8) and described how the outcome relates to the hypothesis of the study (*k* = 27, 87%; item 7). In this category, less frequently reported items described why the primary outcome was relevant to stakeholders (*k* = 2, 6%; item 9), and which stakeholders were actively involved in selection of the outcome (*k* = 2, 6%; item 10).

### How: the way the outcome is measured

Overall, items pertaining to the way the outcome was measured were reported poorly by geriatric depression trials. Although all trials (*k* = 31, 100%) described the OMI used, less than half (*k* = 15, 48%; item 13) included details regarding instrument scaling and scoring. No trial (*k* = 0, 0%; item 15) specified a recall period for outcome assessment. Thirty of the 31 included trials could be assessed for reporting on measurement properties, as the primary outcome for one RCT was provider treatment adherence, which does not have measures of validity, reliability, etc. Only nine studies (30%; item 19) described the validity of the OMI in individuals similar to the study sample, with 17% of trials (*k* = 5; item #0) justifying validity of the OMI in the study setting. Four RCTs (13%; item 22) fully reported reliability of the OMI in a relevant study sample, with even fewer studies (*k* = 2, 7%; item 23) describing reliability of the OMI in the specified study setting. Only a paucity of trials explicitly described responsiveness of the OMI used in the study (*k* = 3, 10%; item 24) or the feasibility (*k* = 2, 7%; item 25), acceptability and/or burden of the OMI in the study sample (*k* = 1, 3%; item 26).

### Who: source of information of the outcome

Descriptions related to the identity and number of outcome assessors were fully reported by just over half of the included trials (*k* = 16, 52%; item 29). However, justification regarding the choice of outcome assessors (*k* = 3, 10%; item 30) and trial-specific training required for outcome assessors (*k* = 8, 26%; item 32) were less frequently reported.

### Where: assessment location and setting of the outcome

The location of outcome assessment was reported by 97% of included studies (*k* = 30; item 34). However, descriptions of the setting of outcome assessment (i.e. clinic, home, other) were reported by 61% of RCTs (*k* = 19, 61%; item 35), with no trial justifying why the outcome setting was suitable for the study sample (*k* = 0, 0%; item 36).

### When: timing of measurement of the outcome

Every included study described the timing and frequency of outcome assessment (*k* = 31, 100%; item 37); however, only 32% of studies (*k* = 10; item 38) provided justification for timing of outcome measurement.

### Outcome data management and analyses

Overall, geriatric depression trials demonstrated good reporting of items pertaining to outcome data management and analyses. All trials (*k* = 31, 100%) described the outcome analysis population (item 39), the unit of analysis of the outcome (item 40), the outcome analysis metric (item 41), the method of aggregation for outcome data (item 42), the statistical methods/significance tests used in analysis (item 43) and the time period for outcome analysis (item 47).

There was variability in the description of items pertaining to outcome management, with between 3 and 29% of items being fully reported by RCTs (items 48–50). Less than a third of studies (*k* = 9, 29%; item 48) described the outcome data, assessment process and analysis for participants who discontinued or deviated from the assigned interventional protocol.

### Missing outcome data

Of the RCTs, 46% or more described how much data was missing, described reasons for missingness in each study arm and explained the statistical methods used to handle missing outcome data (items 52–54). However, only 15% of studies (*k* = 4; item 55) provided justification for the methods used to handle missing data, which was the least frequently reported item in this category.

### Outcome interpretation

Although every study reported an interpretation of outcome data in relation to clinical outcomes (*k* = 31, 100%; item 57), only a paucity of RCTs (*k* = 6, 23%; item 58) discussed the impact of missing outcome data on the interpretation of findings.

## Discussion

Our study found that comprehensiveness of primary outcome reporting in geriatric depression trials published between 2011 and 2021 was variable and mostly insufficient. Notably, the level of detail and descriptions of primary end-points were inconsistent, which impedes full comprehension of markers used to indicate intervention effectiveness. Overall, less than half of the reporting items from the checklist of 58 items were fully reported by trials. Furthermore, outcome reporting was relatively stable and did not improve over the 10-year period. Items that described analysis of the primary outcome were generally fully reported, whereas those that detailed how the end-point was measured were only fully reported in 17% of included trials.

The reporting of outcomes must be conducted in a comprehensive manner, i.e. with sufficient detail to permit full understanding of an end-point, to facilitate transparency of information about the trial from design stage, through to conduct and outcome assessment.^41^ Conversely, variability in outcome reporting, including reporting of insufficient details to permit full understanding of any aspect of a trial's end-point measures, impedes the comparison and synthesis of findings. In particular, this creates difficulty in translating research findings into evidence synthesis products, such as systematic reviews and meta-analyses, consequently reducing their ability to be utilised in clinical decision-making.^16^ Below, we discuss potential reasons for our findings, and implications for pertinent stakeholders, which should be considered in the interpretation, replicability and synthesis of geriatric depression trials.

### Overview of outcome reporting

Although we observed variability in primary outcome reporting across geriatric depression RCTs, it should be noted that several items on our checklist were well-reported across trials. Reporting elements which were well-reported described the timing and frequency of outcome assessment and analyses. Specifically, all trials in our study described the outcome analysis population, unit of analysis, outcome analysis metric, method of aggregation, statistical methods for analysis and the time period for outcome analysis. Our findings also echo those of a recently conducted study on primary outcome reporting in adolescent depression trials.^34^ These results may be attributed to the CONSORT reporting guidelines.^36^ In particular, timing of outcome assessment and outcome analysis represent iterations of items present in the CONSORT reporting guideline, which has been widely used, and is considered the current gold standard for reporting findings from clinical trials.^36^ Although prior research has demonstrated that the CONSORT guideline facilitates comprehensiveness in reporting practices for RCTs,^42–44^ our study highlights that deficiencies in outcome reporting still remain. The general guidance in outcome reporting provided by the original CONSORT statement^36^ may be insufficient to fully ensure full comprehensiveness of reporting practices. Consequently, the recently developed CONSORT-Outcomes checklist^35^ may facilitate standardisation of reporting outcome-specific information in future geriatric depression trials, among other fields.

Our findings indicated that only a paucity of included trials detailed measurement properties of the OMI (i.e. validity, reliability, feasibility), which varied from 3 to 30%. Evaluation of depression symptom severity and/or depression treatment response are subjective health outcomes directly reported by patients, and considered latent constructs that are unable to be measured directly. Thus, psychometric scales are used as OMIs in geriatric depression and psychiatry research at large.^45^ Despite the extensive use of these scales, however, one cannot assume that different OMIs are equally valid in assessing an outcome. A content analysis by Fried^46^ demonstrated only a mean moderate overlap (Jaccard index: 0.41 (average coefficient of overlap across all scales); range: 0 (no overlap) to 1 (complete overlap)) between common OMIs used in depression research.^46,47^ Thus, it is particularly important to report validity, reliability and other measurement properties, to evaluate whether particular OMIs are able to assess such constructs in a valid and reliable manner for the target population in clinical trials. Similarly, a recent review has demonstrated the necessity of including details on measurement properties of OMIs, to communicate the validity of results obtained from using a particular measurement tool, thereby further facilitating understanding of trial outcomes.^39^

### Strengths and limitations

Our assessment was conducted in a systematic manner, and employed methodology used in another study that examined reporting in adolescent depression trials.^34^ Specifically, two trained reviewers performed reporting assessments independently and in duplicate, using a consensus-based approach to resolve discrepancies.

However, our study is not without its limitations. First, we focused on RCTs that specified a single, discernible primary outcome, thereby excluding trials with multiple primary outcomes or those unclear in specifying a primary outcome. Additionally, we included pilot and feasibility studies, whose outcomes are not powered to detect effectiveness.^33^ Thus, our study findings may not reflect the true state of primary outcome reporting in full-scale geriatric MDD trials, particularly in the case of selective reporting in trials with multiple primary outcomes, as evidenced by prior research.^48^ Second, we assessed published trials from 2011 to 2021, thereby excluding unpublished studies or RCTs published outside this period. Nonetheless, given that the trials included in our study spanned a 10-year timeframe, we believe that this is sufficient to assess primary outcome reporting in geriatric depression trials.^49^ Third, the distinction between the categories ‘fully reported’ and ‘partially reported’ may be susceptible to subjectivity in assessment. However, this risk was mitigated by conducting assessment by two reviewers independently and in duplicate (A.O., K.J.), who used a training guide with descriptions and examples of scoring categories, which was developed by methodological experts (M.R., L.T.). Fourth, our 58-item checklist has not been validated, as our study was conducted before publication of the CONSORT-Outcomes guideline,^35^ and all items may not be equally relevant in reporting assessment. However, this checklist has been used in a prior study to assess outcome reporting^34^ and in the development of the eventual CONSORT-Outcomes guideline,^35^ with overlap between items in both checklists.

### Implications for patients, caregivers and clinicians

Two of our findings in particular pose implications for stakeholders of geriatric depression trials: notably, the rationale for primary outcome selection and consideration of meaningful change.

First, the rationale for classifying an outcome as primary was reported by only 23% of trials, suggesting limited consideration of why a particular outcome is used to indicate treatment success. Given that there is an overall lack of consensus about which outcomes are important to measure in a clinical trial for geriatric depression,^29^ it is unsurprising that the rationale for selecting an outcome as a primary indicator of effectiveness is likewise poorly reported. This finding has important implications for patients, caregivers and clinicians. Knowledge of the trial's primary aims and, consequently, clarity in the rationale for outcome selection, would facilitate patient and caregiver understanding of the relevance of the outcome as a marker of treatment success, particularly when the outcome assessed is meaningful to them.^34^ Requirements for reporting the rationale for outcome selection (i.e. through the CONSORT-Outcomes checklist)^35^ may potentially encourage trialists to include primary outcomes that reflect intervention effectiveness in accordance with patient and caregiver perspectives, such as improvements in social functioning, as identified through prior research.^50,51^ Furthermore, an explanation as to why a particular end-point was selected for assessment in a trial would increase its selection in other trials, thereby facilitating the comprehension and comparison of findings between trials through aggregation of results in meta-analyses, and consequently improve evidence-based decision-making.

Second, only 23% of trials in our study justified the criteria for meaningful change, i.e. the minimal important change (MIC) or the minimally clinically important difference. The MIC is a respondent-centred indicator of treatment success that highlights the smallest change on an OMI between two time points that may be considered clinically meaningful.^52^ When fully reported, the MIC has the potential to provide meaningful context and guidance for clinical decision-making, as it constitutes a good or poor outcome, which may therefore be used to infer intervention effectiveness in a clinical trial.^53^ Our finding that only a few trials reported the MIC suggests that determining what constitutes a good or poor outcome is currently based on statistical significance (i.e. mean differences between intervention groups on OMIs), with little regard for what meaningful change would represent to patients, caregivers and clinicians.^54^ Notably, the MIC may be determined with an anchor-based approach, which provides an opportunity for engagement of older adults with depression and their caregivers, and is reflective of the increased movement toward inclusion of patients in health research.^55^ An anchor-based MIC is considered ‘a threshold for a minimal within-person change over time above which patients perceive themselves importantly changed’.^56^ The MIC may be calculated for different respondent groups (patients, caregivers, clinicians) and, when reported in the published report of a clinical trial, serve as binary indicator(s) demonstrating intervention effectiveness. Furthermore, clinicians may utilise established MIC thresholds in their practice when discussing interventions and expected outcomes with patients. Our study therefore highlights the need for determination and reporting of MIC thresholds for OMIs in geriatric depression trials, to extend our understanding of intervention effectiveness beyond mere statistical significance into critical evaluations of whether clinically meaningful change has been achieved for older adults with depression.

### Suggestions for journals

Our study revealed a consistent lack of comprehensive outcome reporting over a 10-year period, which echoes findings from the review on reporting in adolescent depression trials.^34^ Prior research has demonstrated that journal endorsement of CONSORT guidelines are beneficial in improving reporting of RCTs.^42–44^ Given that our study has revealed deficiencies in outcome reporting, in particular, the rationale for outcome selection and definition of clinically meaningful change, journals are recommended to incorporate the CONSORT-Outcomes guideline for reporting outcomes in published trials in the editorial process. Specifically, journals may endorse the CONSORT-Outcomes statement, recommend authors and peer reviewers to follow these guidelines when preparing materials or reviewing manuscripts for publication and/or require submission of the CONSORT-Outcomes checklist by authors.^43^

In conclusion, we found substantial variability in the reporting of primary outcomes across published geriatric depression RCTs. Omission of key details regarding trial outcomes may impede interpretation, replicability and eventual aggregation of trials through knowledge synthesis products that inform clinical guidelines and guide evidence-based decision-making. There is a need for trialists to understand patient perspectives on clinically meaningful outcomes in geriatric depression, and to adhere to outcome reporting guidelines such as the CONSORT-Outcomes statement, when reporting findings from geriatric MDD RCTs.

## Supporting information

Rodrigues et al. supplementary material 1 Rodrigues et al. supplementary material

Rodrigues et al. supplementary material 2 Rodrigues et al. supplementary material

Rodrigues et al. supplementary material 3 Rodrigues et al. supplementary material

Rodrigues et al. supplementary material 4 Rodrigues et al. supplementary material

## Data Availability

The authors confirm that the data supporting the findings of this study are available within the article and/or its supplementary materials.
