# Supplementary material for: Primary outcome reporting in clinical trials for older adults with depression
Source: BJPsych Open. 2024 Mar 7;10(2):e60. doi: 10.1192/bjo.2023.650 (PMC10951853; doi:10.1192/bjo.2023.650)
Supplement: Rodrigues et al. supplementary material 3 — Rodrigues et al. supplementary material [file S2056472423006506sup003.pdf]

Primary outcome reporting in clinical trials for older adults with depression

Additional File C

Table of Contents

Table C1. Framework used to classify outcomes reported in included trials. 2

References for included trials (*n*=31). 3

Table C1. Framework used to classify outcomes reported in included trials.

Core area [a]	Outcome domain [a]	Outcome subdomain [a]	Outcome term [a]	Definition	Patient-important outcome major domain [b]	Patient-important outcome subdomain [b]	Patient-important outcome term [b]	Outcome type [c]	Number of trials which reported outcome term as single, discernable primary outcome (%)
Physiological/ Clinical	Psychiatric outcomes	Depression outcomes	Depression relapse	The opposite of remission. Assessment of the continual presence of the symptoms of depression. Often a binary measure for meeting a threshold value assessed at the end of the trial.	None	None	None	Patient-reported	1 (3%)
			Depression remission	The extent to which symptoms of depression have been resolved by the intervention/comparator. Often a binary measure for meeting a threshold value assessed at the end of the trial (no threshold specified).	Other	Medication efficacy	Goal of the treatment: recovery	Patient-reported	1 (3%)
			Depression treatment response	The extent to which symptoms of depression have been resolved by the intervention/comparator. Often a difference (continuous measure) comparing baseline and final scores on measures for participants.	Other	Medication efficacy	Goal of the treatment: recovery	Patient-reported	12 (39%)
			Depressive symptom severity	The extent to which symptoms of depression affect the patient, and may range from 'mild' to 'severe'. Often a difference (continuous measure) comparing final scores between arms.	Other	Medication efficacy	Less residual symptoms	Patient-reported	15 (48%)
Life Impact	Delivery of care	N/A	Provider treatment adherence	Compliance of primary care physicians in implementing guideline recommendations for management of older adults with depression (management trial).	None	None	None	Clinician-reported	1 (3%)
Resource Use	Economic	N/A	Cost-effectiveness of study interventions	Economic analysis comparing the relative costs and outcomes of different interventions / comparators used in the trial.	None	None	None	Other	1 (3%)

Notes:

a: Outcome terms were mapped onto three existing frameworks. First, all outcome terms were assigned to the taxonomic classification system proposed by Dodd et al. (2018), which comprise five “core areas”: physiological/clinical, life impact, resource use, adverse events, and death, comprised of suggested domains and with the option for the user to add subdomains. We classified outcomes using this framework in our previous study (Rodrigues et al., 2023).

<u>Additional note:</u> *Although there are five core areas, no outcomes in our current study fell under the areas of “adverse events” or “death”.*

b: Second, all outcome terms were mapped to a list of patient-important outcomes identified by a recent global survey (Chevance et al., 2020), which categorized outcome terms into three major domains: symptom-related, improvement of functioning, and other with subdomains.

c: Third, we classified outcome types: clinical (i.e., physical examinations), surrogate markers (i.e., neurobiomarkers), reported by patients, carers or clinicians (i.e., psychometric scales or adherence), or other (i.e., economic analyses) (Papakostas, 2012).

References:

Chevance A, Ravaud P, Tomlinson A, Le Berre C, Teufer B, Touboul S, et al. Identifying outcomes for depression that matter to patients, informal caregivers, and health-care professionals: qualitative content analysis of a large international online survey. The Lancet Psychiatry 2020;7:692–702.

Dodd S, Clarke M, Becker L, Mavergames C, Fish R, Williamson PR. A taxonomy has been developed for outcomes in medical research to help improve knowledge discovery. J Clin Epidemiol 2018;96:84–92.

Papakostas GI. Surrogate markers of treatment outcome in major depressive disorder. Int J Neuropsychopharmacol 2012;15:841–54.

Rodrigues M, Syed Z, Dufort A, Sanger N, Ghiassi P, Sanger S, Panesar B, D’Elia A, Parpia S, Samaan Z, Thabane L. Heterogeneity across outcomes reported in clinical trials for older adults with depression: a systematic survey. Journal of Clinical Epidemiology 2023;157:59-73.

References for included trials (*n*=31).

1. Banerjee S, Hellier J, Dewey M, Romeo R, Ballard C, Baldwin R, Bentham P, Fox C, Holmes C, Katona C, Knapp M. Sertraline or mirtazapine for depression in dementia (HTA-SADD): a randomised, multicentre, double-blind, placebo-controlled trial. The Lancet. 2011 Jul 30;378(9789):403-11.
2. Brody BL, Field LC, Roch-Levecq AC, Moutier CY, Edland SD, Brown SI. Treatment of Depression Associated with Age-related Macular Degeneration in a Double-Blind Randomized Controlled Study. Annals of clinical psychiatry: official journal of the American Academy of Clinical Psychiatrists. 2011 Nov;23(4):277.
3. Chen YM, Huang XM, Thompson R, Zhao YB. Clinical features and efficacy of escitalopram treatment for geriatric depression. Journal of International Medical Research. 2011 Oct;39(5):1946-53.
4. Rondanelli M, Giacosa A, Opizzi A, Pelucchi C, La Vecchia C, Montorfano G, Negroni M, Berra B, Politi P, Rizzo AM. Long chain omega 3 polyunsaturated fatty acids supplementation in the treatment of elderly depression: effects on depressive symptoms, on phospholipids fatty acids profile and on health-related quality of life. The journal of nutrition, health & aging. 2011 Jan;15(1):37-44.
5. Tajalizadekhoob Y, Sharifi F, Fakhrzadeh H, Mirarefin M, Ghaderpanahi M, Badamchizade Z, Azimipour S. The effect of low-dose omega 3 fatty acids on the treatment of mild to moderate depression in the elderly: a double-blind, randomized, placebo-controlled study. European archives of psychiatry and clinical neuroscience. 2011 Dec;261(8):539-49.
6. Cheng ST, Chow PK, Edwin CS, Chan AC. Leisure activities alleviate depressive symptoms in nursing home residents with very mild or mild dementia. The American Journal of Geriatric Psychiatry. 2012 Oct 1;20(10):904-8.
7. Fields C, Drye L, Vaidya V, Lyketsos C, ADAPT Research Group. Celecoxib or naproxen treatment does not benefit depressive symptoms in persons age 70 and older: findings from a randomized controlled trial. The American Journal of Geriatric Psychiatry. 2012 Jun 1;20(6):505-13.
8. Preschl B, Maercker A, Wagner B, Forstmeier S, Baños RM, Alcañiz M, Castilla D, Botella C. Life-review therapy with computer supplements for depression in the elderly: a randomized controlled trial. Aging & mental health. 2012 Nov 1;16(8):964-74.
9. Heun R, Ahokas A, Boyer P, Giménez-Montesinos N, Pontes-Soares F, Olivier V, Agomelatine Study Group. The efficacy of agomelatine in elderly patients with recurrent major depressive disorder: a placebo-controlled study. The Journal of clinical psychiatry. 2013 Jun 15;74(6):5943.
10. Katila H, Mezhebovsky I, Mulroy A, Berggren L, Eriksson H, Earley W, Datto C. Randomized, double-blind study of the efficacy and tolerability of extended release quetiapine fumarate (quetiapine XR) monotherapy in elderly patients with major depressive disorder. The American Journal of Geriatric Psychiatry. 2013 Aug 1;21(8):769-84.
11. Robinson M, Oakes TM, Raskin J, Liu P, Shoemaker S, Nelson JC. Acute and long-term treatment of late-life major depressive disorder: duloxetine versus placebo. The American Journal of Geriatric Psychiatry. 2014 Jan 1;22(1):34-45.
12. Bruce ML, Raue PJ, Reilly CF, Greenberg RL, Meyers BS, Banerjee S, Pickett YR, Sheeran TF, Ghesquiere A, Zukowski DM, Rosas VH. Clinical effectiveness of integrating depression care management into medicare home health: the Depression CAREPATH Randomized trial. JAMA internal medicine. 2015 Jan 1;175(1):55-64.
13. Imai H, Furukawa TA, Okumiya K, Wada T, Fukutomi E, Sakamoto R, Fujisawa M, Ishimoto Y, Kimura Y, Chen WL, Tanaka M. Postcard intervention for depression in community-dwelling older adults: A randomised controlled trial. Psychiatry research. 2015 Sep 30;229(1-2):545-50.
14. McCusker J, Cole MG, Yaffe M, Strumpf E, Sewitch M, Sussman T, Ciampi A, Lavoie K, Platt RW, Belzile E. A randomized trial of a depression self-care toolkit with or without lay telephone coaching for primary care patients with chronic physical conditions. General Hospital Psychiatry. 2015 May 1;37(3):257-65.

15. van Beljouw IM, van Exel E, van de Ven PM, Joling KJ, Dhondt TD, Stek ML, van Marwijk HW. Does an outreaching stepped care program reduce depressive symptoms in community-dwelling older adults? A randomized implementation trial. The American Journal of Geriatric Psychiatry. 2015 Aug 1;23(8):807-17.
16. Aakhus E, Granlund I, Odgaard-Jensen J, Oxman AD, Flottorp SA. A tailored intervention to implement guideline recommendations for elderly patients with depression in primary care: a pragmatic cluster randomised trial. Implementation Science. 2015 Dec;11(1):1-5.
17. Bosanquet K, Adamson J, Atherton K, Bailey D, Baxter C, Beresford-Dent J, Birtwistle J, Chew-Graham C, Clare E, Delgadillo J, Ekers D. CollAborative care for Screen-Positive EldeRs with major depression (CASPER plus): a multicentred randomised controlled trial of clinical effectiveness and cost-effectiveness. Health Technology Assessment (Winchester, England). 2017 Nov;21(67):1.
18. Hummel J, Weisbrod C, Boesch L, Himpler K, Hauer K, Hautzinger M, Gaebel A, Zieschang T, Fickelscherer A, Diener S, Dutzi I. AIDE–acute illness and depression in elderly patients. Cognitive behavioral group psychotherapy in geriatric patients with comorbid depression: A randomized, controlled trial. Journal of the American Medical Directors Association. 2017 Apr 1;18(4):341-9.
19. Chang KJ, Hong CH, Roh HW, Lee KS, Lee EH, Kim J, Lim HK, Son SJ. A 12-week multi-domain lifestyle modification to reduce depressive symptoms in older adults: a preliminary report. Psychiatry Investigation. 2018 Mar;15(3):279.
20. Emsley R, Ahokas A, Suarez A, Marinescu D, Dóci I, Lehtmets A, Milanova V, Lee MS, Didi R, Araszkiewicz A, Sulaiman AH. Efficacy of tianeptine 25-50 mg in elderly patients with recurrent major depressive disorder: an 8-week placebo-and escitalopram-controlled study. The Journal of clinical psychiatry. 2018 Jul 3;79(4):21006.
21. Molassiotis A, Suen L, Lai C, Chan B, Wat KH, Tang J, To KL, Leung CO, Lee S, Lee P, Chien WT. The effectiveness of acupressure in the management of depressive symptoms and in improving quality of life in older people living in the community: a randomised sham-controlled trial. Aging & Mental Health. 2020 Jun 2;24(6):1001-9.
22. Ochs-Ross R, Daly EJ, Zhang Y, Lane R, Lim P, Morrison RL, Hough D, Manji H, Drevets WC, Sanacora G, Steffens DC. Efficacy and safety of esketamine nasal spray plus an oral antidepressant in elderly patients with treatment-resistant depression—TRANSFORM-3. The American Journal of Geriatric Psychiatry. 2020 Feb 1;28(2):121-41.
23. Berk M, Agustini B, Woods RL, Nelson MR, Shah RC, Reid CM, Storey E, Fitzgerald SM, Lockery JE, Wolfe R, Mohebbi M. Effects of aspirin on the long-term management of depression in older people: a double-blind randomised placebo-controlled trial. Molecular psychiatry. 2021 Sep;26(9):5161-70.
24. Tomasino KN, Lattie EG, Ho J, Palac HL, Kaiser SM, Mohr DC. Harnessing peer support in an online intervention for older adults with depression. The American Journal of Geriatric Psychiatry. 2017 Oct 1;25(10):1109-19.
25. Burroughs H, Bartlam B, Bullock P, Lovell K, Ogollah R, Ray M, Bower P, Waheed W, Gilbody S, Kingstone T, Nicholls E. Non-traditional support workers delivering a brief psychosocial intervention for older people with anxiety and depression: the NOTEPAD feasibility study. NIHR Journals Library. 2019 Jul 26.
26. Bollmann S, Spies J, Hodzic J, Hohl-Radke F, Brakemeier EL. Feasibility and outcome of an inpatient interpersonal skills group for older adults (65+) with acute or chronic depression: An open pilot study. GeroPsych: The Journal of Gerontopsychology and Geriatric Psychiatry. 2020;33(2):55.
27. Azar AR, Chopra MP, Cho LY, Coakley E, Rudolph JL. Remission in major depression: results from a geriatric primary care population. International journal of geriatric psychiatry. 2011 Jan;26(1):48-55.
28. Drye LT, Martin BK, Frangakis CE, Meinert CL, Mintzer JE, Munro CA, Porsteinsson AP, Rabins PV, Rosenberg PB, Schneider LS, Weintraub D. Do treatment effects vary among differing baseline depression criteria in depression in Alzheimer's disease study±2 (DIADS-2)?. International journal of geriatric psychiatry. 2011 Jun;26(6):573-83.

29. Holman AJ, Serfaty MA, Leurent BE, King MB. Cost-effectiveness of cognitive behaviour therapy versus talking and usual care for depressed older people in primary care. BMC health services research. 2011 Dec;11(1):1-9.
30. Dolberg O, Larsson Lönn S, Kvist K. Factors predicting relapse in elderly patients with major depressive disorder treated with escitalopram in an outpatient setting. Current medical research and opinion. 2014 Jul 1;30(7):1301-7.
31. Zilcha-Mano S, Roose SP, Brown PJ, Rutherford BR. Abrupt Symptom Improvements in Antidepressant Clinical Trials: Transient Placebo Effects or Therapeutic Reality?. The Journal of Clinical Psychiatry. 2018 Dec 4;80(1):20250.
